# Electrotransfer of siRNA to Silence Enhanced Green Fluorescent Protein in Tumor Mediated by a High Intensity Pulsed Electromagnetic Field

**DOI:** 10.3390/vaccines8010049

**Published:** 2020-01-27

**Authors:** Simona Kranjc Brezar, Matej Kranjc, Maja Čemažar, Simon Buček, Gregor Serša, Damijan Miklavčič

**Affiliations:** 1 Department of Experimental Oncology, Institute of Oncology Ljubljana, Zaloška 2, 1000 Ljubljana, Slovenia; skranjc@onko-i.si (S.K.B.); mcemazar@onko-i.si (M.Č.); gsersa@onko-i.si (G.S.); 2 Faculty of Electrical Engineering, University of Ljubljana, Tržaška 25, 1000 Ljubljana, Slovenia; matej.kranjc@fe.uni-lj.si; 3 Faculty of Health Sciences, University of Primorska, Polje 42, 6310 Izola, Slovenia; 4 Department of Cytopathology, Institute of Oncology Ljubljana, Zaloška 2, 1000 Ljubljana, Slovenia; sbucek@onko-i.si; 5 Faculty of Health Sciences, University of Ljubljana, Zdravstvena pot 5, 1000 Ljubljana, Slovenia

**Keywords:** high intensity pulsed electromagnetic field, electrotransfer, enhanced green fluorescent protein, siRNA, mouse melanoma model, tumor

## Abstract

The contactless high intensity pulsed electromagnetic field (HI-PEMF)-induced increase of cell membrane permeability is similar to conventional electroporation, with the important difference of inducing an electric field non-invasively by exposing a treated tissue to a time-varying magnetic field. Due to the limited number of studies in the field of electroporation induced by HI-PEMF, we designed experiments to explore the feasibility of such a contactless delivery technique for the gene electrotransfer of nucleic acids in tissues in vivo. By using HI-PEMF for gene electrotransfer, we silenced enhanced green fluorescent protein (EGFP) with siRNA molecules against EGFP in B16F10-EGFP tumors. Six days after the transfer, the fluorescent tumor area decreased by up to 39% as determined by fluorescence imaging in vivo. In addition, the silencing of EGFP to the same extent was confirmed at the mRNA and protein level. The results obtained in the in vivo mouse model demonstrate the potential use of HI-PEMF-induced cell permeabilization for gene therapy and DNA vaccination. Further studies are thus warranted to improve the equipment, optimize the protocols for gene transfer and the HI-PEMF parameters, and demonstrate the effects of HI-PEMF on a broader range of different normal and tumor tissues.

## 1. Introduction

Gene transfer depends on the effective delivery of nucleic acids (DNA, RNA) into cells or tissue and can be enabled by viral or nonviral delivery methods. One method for the nonviral delivery of genetic material (plasmid DNA, siRNA, and miRNA) into cells is electroporation. Electroporation is generally attributed to the formation of pores immediately after the induced transmembrane voltage exceeds physiological values [1,2]. The use of electroporation as a gene delivery method (gene electrotransfer) was first reported in 1982 [3] and has since reached a broad spectrum of applications in different tissues, such as tumor, muscle, and skin [4,5,6,7]. Electroporation is also used for the delivery of impermeant small molecules, such as dyes and drugs, into cells. Applying electroporation to facilitate intracellular delivery of cytotoxic drugs, such as bleomycin and cisplatin, is known as electrochemotherapy [8]. Besides enabling delivery of genes with immunomodulatory effects or drugs to target cells, gene electrotransfer and electrochemotherapy are able to induce immune response, leading to an antitumor effectiveness [9,10,11]. Over the past several years, electroporation was successfully translated to veterinary and human clinics [12,13,14]. 

Conventional electroporation is achieved by pulsed electric fields established in tissue by the delivery of a train of electric pulses of high voltage via electrodes [15,16]. Depending on the chosen electric field parameters, electroporation can be reversible (when the cell membrane recovers in time), or can be irreversible, which occurs when the applied electric field induces irrecoverable damage to the cell [17]. In addition to electric field parameters, most notably amplitude, pulse duration, and pulse number, other factors influence the efficiency of molecule uptake, e.g., the size and charge of molecules, osmotic pressure (osmolarity of the electroporation buffer), the pH of the buffer, and temperature [18,19]. Furthermore, tissue characteristics such as homogeneity, i.e., cell size and type, the composition of the extracellular matrix, and the degree of vascularization also affect current density and electric field distribution as well as drug uptake [20,21].

In the past, the effects of externally applied pulsed electromagnetic fields and static magnetic fields on cells were studied extensively. It has been demonstrated that relatively low magnetic fields can influence intracellular signal transduction, affect cytoskeletal proteins involved in cell shape modification, and induce changes in the mitochondrial membrane potential [22,23,24]. High intensity pulsed electromagnetic fields (HI-PEMF) were also demonstrated to increase transmembrane molecular transport, presumably through the successful electroporation of the cell membrane [25,26]. Furthermore, HI-PEMFs were successfully used for drug and plasmid DNA delivery in vitro and in vivo [27,28,29]. Thus, HI-PEMF has the potential for achieving cell and tissue electroporation in a noncontact manner.

Due to the limited number of studies in the field of cell membrane permeabilization induced by HI-PEMF, we designed experiments to explore the feasibility of such a contactless delivery technique for the electrotransfer of small nucleic acids in tumors in vivo. For that purpose, the transfection efficiency of small siRNA molecules against the enhanced green fluorescent protein (EGFP) in B16F10 melanoma tumors stably expressing EGFP were measured. HI-PEMF-induced cell permeabilization was proved to facilitate small siRNA molecule uptake in tumors; thus, we provide evidence of its feasibility and effectiveness. The HI-PEMF approach could have an advantage over conventional electroporation since HI-PEMF is noninvasive, contactless, and potentially painless.

## 2. Materials and Methods 

### 2.1. siRNA

siRNA was designed according to Caplen et al. [30] with freely available software from Eurofins Genomics. The EGFP siRNA (sense: 5’-GCAAGCUGACCCUGAAGUUCA, antisense: 5’-UGAACUUCAGGGUCAGCUUGC) was directed against EGFP mRNA. As a control for the specificity of the siRNA construct, we used scramble (SCR) siRNA (sense: 5’-TTGATCGTTTGCTACGCTTTACTTC, antisense: 5’-UUGAUCGUUUGCUACGCUUUACUUC) (Invitrogen, Thermo Fisher Scientific, Carlsbad, CA, USA), which contains the changed nucleotide siRNA sequence for mouse endoglin and shows no significant homology to mouse transcripts according to Basic Local Alignment Search Tool analysis. siRNAs were prepared at a final concentration of 20 µM in 40 µL of siMAX universal buffer (Eurofins Genomics) containing 40 U of RNAase inhibitor (Applied Biosystems by Life Technologies, Thermo Fisher Scientific).

### 2.2. Mice, Tumor Model

Female C57Bl/6JOlaHsd mice (Envigo RMS S.r.l., San Pietro al Natisone, Italy) were maintained in quarantine for 2–3 weeks after purchase. In the experiments, 145 mice were used. They were 8- to 9-weeks-old, they weighed 20–22 g, and were kept at a constant room temperature with a 12 h light cycle. Tumors were implanted subcutaneously into the C57Bl/6 mice in the right flank of the mice by inoculation of a suspension 1 × 10^6^ B16F10-EGFP melanoma cells prepared in 100 µL of PBS. During the injection of siRNA and the delivery of HI-PEMF or square wave electric pulses, mice were anesthetized with inhalation anesthesia in the induction chamber with 2% (v/v) of isoflurane (Isoflurane; Piramal Healthcare UK Limited, London, UK). All animal experimental manipulations were conducted in accordance with the principles and procedures outlined in the guidelines for animal experiments of the EU directives and with permission from the Administration of the Republic of Slovenia for Food Safety, Veterinary and Plant Protection (Republic of Slovenia, The Ministry of Agriculture, Forestry and Food) (permission No: U34401–1/2015/7).

### 2.3. Experimental Setup

**Figure 1:**
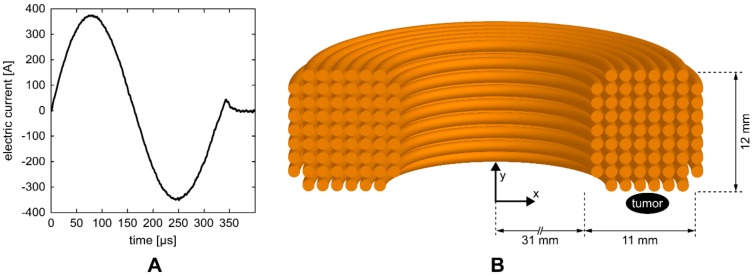
(**A**) One of the electric pulses that were delivered to the coil of the applicator. (**B**) Illustration of the cross-section of the coil with 68 turns. The position of the treated tumor was located at the periphery of the coil just below the middle of the windings.

HI-PEMF was delivered by a custom-made magnetic field pulse generator connected to an applicator consisting of a round coil with 68 turns. The generator supplied the applicator with bipolar electric pulses (Figure 1A) that generated a time-varying HI-PEMF in the vicinity of the coil. As shown in Figure 1B, the applicator was positioned over the treated tissue so that the tumor was located at the periphery of the coil just below the middle of the windings where the induced electric field is the highest. The pulse sequences of the electric current delivered to the applicator consisted of 400 bipolar pulses (Figure 1A) at the repetition frequency of 33 Hz (the application of HI-PEMF lasted a total of 12 s).

For comparison, siRNA electrotransfer (GET) achieved by conventional electroporation was performed. Eight 100 µs long square wave electric pulses at 600 V/cm voltage to distance ratio delivered at 1 Hz using electric pulse generator (Cliniporator™, IGEA s.r.l., Carpi, Italy) were applied by plate electrodes (distance between the electrodes was 6 mm) to the tumors. The application of electric pulses lasted a total of 8 s and were delivered in perpendicular orientation (4 + 4). To achieve good contact between the electrodes and tumor, the area around the tumor (d = 1 cm), on the back of the mouse was shaved and depilated with depilatory cream (Veet^®^ Sensitive Skin, Reckitt Benckiser, UK). Conductive gel (Gel G006 ECO, FIAB, Vicchio, Italy) was applied to the skin just prior to the application of pulses.

### 2.4. Magnetic Field and Induced Electric Field

The magnetic field and induced electric field in the tumor were estimated using the finite element method and the commercial finite element software package COMSOL Multiphysics 5.4 (COMSOL AB, Stockholm, Sweden). The applicator was designed using the Magnetic Fields physics module as a coil with 68 wires separated by an electrical insulator, while the tumor was represented by an elipsoid. As an input, the measured current pulse waveform (Figure 1A) was used and an automated physics-controlled meshing was applied. The average values of the magnetic field and induced electric field in the volume of the tumor were 0.55 T and 14 V/m, respectively. Whereas the maximum values of magnetic field and induced electric field in the tumor were 0.66 T and 23 V/m, respectively.

### 2.5. In Vivo siRNA Gene Silencing in Subcutaneous Tumors

**Figure 2:**
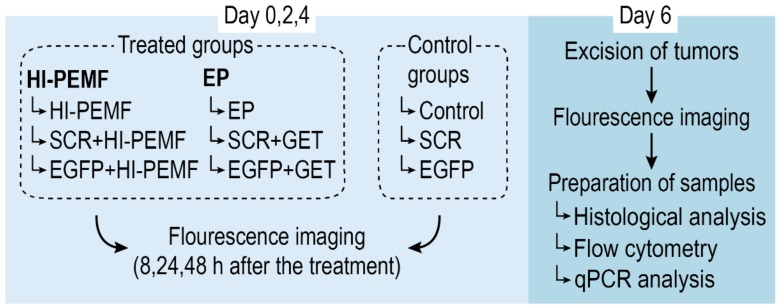
Overview of the treatment protocol for assessment of siRNA gene silencing in subcutaneous tumors. On day 0, mice were randomly divided into treated and control groups. Treated groups were subject to either HI-PEMF or GET (conventional electroporation (EP)) and intratumoral injections of either EGFP siRNA, siMAX universal buffer or SCR siRNA, whereas control groups were subject to injections only. Treatment and injections were performed every second day three consecutive times (on day 0, 2, and 4) followed by fluorescence imaging of the tumors 8, 24, and 48 h after the treatment. On day 6, tumors were excised, imaged, and prepared for histological, flow cytometry, and qPCR analysis.

When the subcutaneous B16F10-EGFP tumors reached a volume of approximately 45 mm^3^, mice were randomly divided into experimental groups (Figure 2): intratumoral injection of siMAX universal buffer with RNAase inhibitor alone (Control) or combined with electroporation induced by HI-PEMF (HI-PEMF) or combined with conventional electroporation (EP), intratumoral injection of control SCR siRNA alone (SCR) or combined with electroporation induced by HI-PEMF (SCR+HI-PEMF) or combined with conventional EP (SCR+GET), and intratumoral injection of EGFP siRNA alone (EGFP) or combined with electroporation induced by HI-PEMF (EGFP+HI-PEMF) or combined with conventional EP (EGFP+GET). Injections were performed on each day of the therapy. HI-PEMF and GET were performed every second day three consecutive times (on days 0, 2, and 4) 10 min after the slow injection of 40 µL of EGFP or SCR siRNA.

### 2.6. Total mRNA Extraction and Quantitative Reverse Transcription-Polymerase Chain Reaction (qRT-PCR) Analysis

RNA extraction and qRT-PCR analysis were used to determine the EGFP expression level and the extent of silencing EGFP at the mRNA level two days after the last siRNA electrotransfer (Figure 2). At that time, animals were sacrificed, tumors were excised and divided into two equal parts; one part was used for flow cytometry analysis (described in the section below) and the other part was used for qRT-PCR analysis. For the qRT-PCR analysis, tumor samples were snap-frozen in liquid nitrogen and later homogenized in a mortar with a pestle and total RNA was extracted from B16F10-EGFP tumors using the peqGOLD Total RNA kit (PEQLAB, VWR™, Life Science, Leuven, Belgium). RNA concentration and purity were determined spectrophotometrically (Epoch Microplate Spectrophotometer; Take3TM Micro-Volume Plate, BioTek, Bad Friedrichshall, DE). Total RNA (2000 ng) was reversely transcribed to cDNA by SuperScript VILO cDNA Synthesis Kit (Invitrogen, Thermo Fisher Scientific, Carlsbad, USA) and mixtures of transcribed cDNA diluted 10-fold were used as templates in qRT-PCR. The qRT-PCR was performed with Mix TaqMan Gene Expression Master Mix (Applied Biosystems, Thermo Fisher Scientific) and TaqMan Gene Expression Assay (Applied Biosystems, Life Technologies) for EGFP (Mr04097229_mr) and murine GAPDH (Mm99999915_g1), as an internal control. The reaction was performed on Quant Studio 3 (Applied Biosystems, Life Technologies) and the results were analyzed with Quant Studio^®^ Design & Analysis Software v1.1 (Applied Biosystems, Life Technologies). The optimized thermal cycling conditions were as follows: activation of uracil-DNA glycosylase (2 min at 50 °C), hot start activation of AmpliTaq Gold Enzyme (10 min at 95 °C), 40 cycles of denaturation (15 s at 95 °C), annealing and extension (1 min at 60 °C). The EGFP mRNA expression levels in tumors were presented as the threshold cycle value (Ct). The relative quantification of cDNA expression was normalized to glyceraldehyde 3-phosphate dehydrogenase (GAPDH) by the 2^−ΔΔCt^ method, calculated via the QuantStudio 3 software (Applied Biosystems, Life Technologies).

### 2.7. Flow Cytometry

For the quantification of the silencing of EGFP at the protein level, flow cytometry analysis two days after the last siRNA electrotransfer was performed (Figure 2). The second half of tumor samples were cut into small pieces and incubated in 5 mL of HBSS (Gibco, Thermo Fisher Scientific) solution containing collagenase II (10 mg/mL, Worthington Biochemical Corporation, Lakewood, USA), DNase I (2 U/mL, Invitrogen, Thermo Fisher Scientific) for 45 min with regular shaking (160 rpm) at 37°C. Cell suspensions were then strained through 50-μm nylon strainers (Sysmec Partec GmbH, Görlitz, Germany) and centrifuged for 5 min at 400 g. Obtained cell pellets were resuspended in 500 μL of PBS with 5% of fetal bovine serum (Gibco, Thermo Fisher Scientific) and stained with the DNA binding dye propidium iodide (PI, 50 µg/mL, Sigma Aldrich, Buchs, Switzerland). Then, fluorescence analysis of the samples was performed using a FACSCanto II flow cytometer (BD Biosciences, San Jose, CA). The viable, PI cell population was gated from the biparametric linear plot, defined by forward and side scatter, to eliminate debris. Furthermore, the histograms of gated cells against the observed fluorescence intensity were recorded (software: BD FACSDiva V6.1.2). The median fluorescence intensity values for EGFP were determined for each experimental group and normalized to the median fluorescence intensity of the untreated cells.

### 2.8. Noninvasive Stereomicroscope Fluorescence Imaging

Tumors were monitored before and after the therapy at least once per day with a stereomicroscope at room temperature, which was carried out using a Carl Zeiss SteREO Lumar V12 fluorescence stereomicroscope equipped with a NeoLumar S 0.8 × objective and an AxioCam MRc5 digital camera (all from Carl Zeiss, Jena, Germany). This equipment allowed the observation of the EGFP expression in the same animal for several days. The animals were anesthetized with isoflurane inhalation anesthesia (2%, v/v) and placed on a custom-designed holder. In the experiments using EGFP siRNA at 8 h, 24 h, and 48 h after treatment, bright-field images and images obtained with a GFP filter of the tumors were taken to observe the changes in the EGFP expression in the cells directly through the skin. Thereafter, the animals were humanely sacrificed, and the target tissues were carefully removed and imaged under bright-field conditions and a GFP filter to determine the precise position of transfection. The exposure time was set at 1.5 s with no binning. The EGFP fluorescence from the tumor was quantitatively evaluated at the measured time points. From the bright-field images acquired at 10 × magnification for tumors, the target tissue was located and manually gated to determine the region of interest. For the images taken with the GFP filter, a suitable threshold was applied, and the fluorescence intensity in the whole area of the tumor, i.e., the region of interest determined from bright-field images, was determined with image analysis software (AxioVision). Aiming to achieve the best conditions for in vivo imaging in the treated tissue, the area around the tumor (d = 1 cm) on the back of the mouse was shaved and depilated. In addition, immediately after excision and imaging, tumors were used for further flow cytometry analysis, as described in the section above, or histology analysis, as described in the section below.

### 2.9. Histology

Histology analysis was performed in tumors after repetitive siRNA EGFP electrotransfer by HI-PEMF application or EP, in order to determine the effect on cell proliferation, apoptosis, necrosis, and immune cell infiltration. Briefly, tumors (untreated-control, treated: HI-PEMF, EP, siRNA EGFP electrotransfer using HI-PEMF (EGFP+HI-PEMF) or EP (EGFP+GET) were excised on day 6 (2 days after the third siRNA electrotransfer with HI-PEMF or EP). The tumors were fixed in IHC zinc fixative (BD Biosciences, San Diego, CA, USA), embedded in paraffin blocks and cut to 2-μm thick consecutive sections. The first tumor section was stained with hematoxylin and eosin (H&E) to estimate the percent of necrotic tumor area, the other three sections were stained immunohistochemically to determine the percent of apoptosis, proliferation, immune cells (granzyme B). The proliferation rate was detected with antibodies against Ki-67 at dilution 1:1200, apoptotic cells were determined with antibodies against cleaved Caspase-3 (Ca-3, Cell signaling Technology, Danvers, MA, USA) at dilution 1:1500. In addition, possible immune cell infiltration (CTL and NK) was detected with antibodies against Granzyme B (ab4059, Abcam) at dilution 1:1250. These primary antibodies were then detected with a peroxidase-conjugated streptavidin-biotin secondary antibody (Rabbit specific HRP/DAB detection IHC kit, ab64261, Abcam) and counterstained with hematoxylin, as described previously [31]. Images of five viable parts in each tumor section were captured by DP72 CCD camera (Olympus, Hamburg, Germany) connected to BX-51 microscope (Olympus, Hamburg, Germany) under 60× magnification. The number of stained brown (positive) and stained blue (negative) cells were counted using CellSens Dimension Software (Olympus) and presented as the percent of the positive cells for each image (apoptosis, proliferation, immune cells). In addition, a necrotic area in the tumors was measured with the same software.

### 2.10. Statistical Analysis

All data were tested for a normal distribution with the Shapiro–Wilk test. A t-test and one-way analysis of variance followed by a Holm–Sidak test were used to evaluate the differences between the experimental groups. A *p*-value less than 0.05 was considered significant. SigmaPlot software (Systat Software, Chicago, IL, USA) was used for statistical analysis and graphical presentation.

## 3. Results

### 3.1. Electrotransfer of siRNA Against EGFP Using HI-PEMF Efficiently Silences EGFP Expression at mRNA and Protein Level, In Vivo

**Figure 3:**
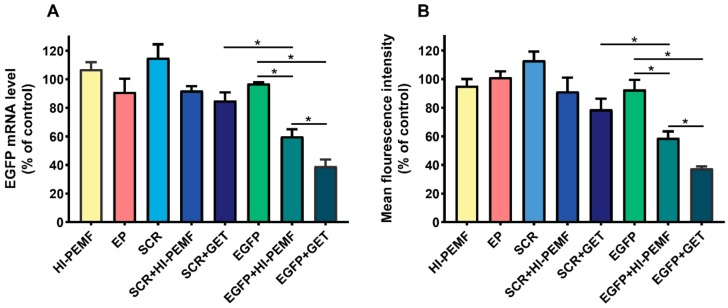
In vivo reduced expression of EGFP mRNA level and protein EGFP level after silencing in B16F10 tumors stably expressing EGFP. Tumors were treated with an intratumoral injection of either siMAX universal buffer (Control group, *n* = 6) or EGFP siRNA (EGFP group, *n* = 8) or SCR siRNA alone (SCR group-scramble, noncoding siRNA, *n* = 8), or with electrotransfer after electroporation by a high intensity pulsed electromagnetic field (HI-PEMF, *n* = 8) or conventional electroporation settings (EP, *n* = 6), as well as with a combination of siRNA electrotransfer by HI-PEMF (*n* = 8) or conventional EP (GET, *n* = 8). (**A**) mRNA level of EGFP in B16F10 tumors stably expressing EGFP determined by qRT-PCR analysis. The data represent the mean and standard error of the mean. The mRNA levels of EGFP in tumors of all experimental groups were normalized to the mRNA level of the untreated group. (**B**) EGFP protein level in B16F10 tumors stably expressing EGFP determined by flow cytometry. The fluorescence intensity of EGFP in tumors of all experimental groups was normalized to the fluorescence intensity of the untreated group. The data represent the mean and standard error of the mean. *- indicate statistically significant differences, *p* < 0.05.

Efficient silencing of EGFP after electrotransfer by HI-PEMF was demonstrated by qRT-PCR analysis of the EGFP expression in B16F10 tumors stably expressing EGFP 2 days after the third electrotransfer. EGFP mRNA levels were significantly reduced after HI-PEMF and conventional gene electrotransfer. The level of EGFP mRNA was reduced for 40% after electrotransfer by HI-PEMF and for 61% by conventional GET (Figure 3A). Furthermore, the EGFP protein level in B16F10 tumors stably expressing EGFP was determined 2 days after the last electrotransfer with flow cytometry. The expression of EGFP at the protein level was significantly decreased after HI-PEMF and conventional gene electrotransfer. The level of EFGP protein was reduced by 41% after using HI-PEMF for electrotransfer and for 63% after using GET (Figure 3B). The silencing effect at the mRNA and protein level after conventional electrotransfer was significantly higher than after HI-PEMF. However, conventional or HI-PEMF electrotransfer with SCR siRNA had no significant effect on the EGFP mRNA and protein level (Figure 3A,B).

### 3.2. Silencing of EGFP after Noninvasive Electroporation by HI-PEMF, Imaging In Vivo

**Figure 4:**
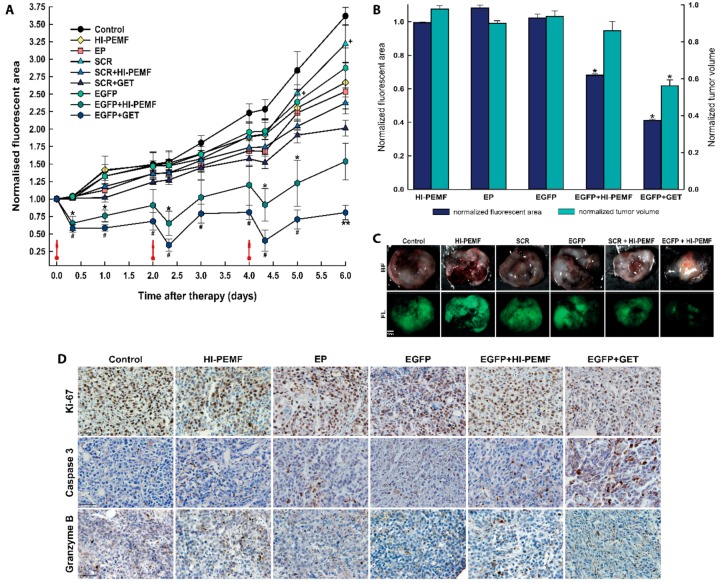
In vivo silencing of enhanced green fluorescent protein (EGFP) with EGFP siRNA using electrotransfer by a high intensity pulsed electromagnetic field (HI-PEMF) or conventional electroporation (EP) in B16F10 tumors stably expressing EGFP. (**A**) Imaging with a fluorescence stereomicroscope was used for the quantification of the time-lapse fluorescence of B16F10 EGFP tumors. For each animal, the fluorescent area of the tumor was measured at different time points and normalized to the value of the fluorescent area measured just before the first treatment on day 0. B16F10 EGFP tumors were treated with an intratumoral injection of either siMAX universal buffer (Control group, *n* = 6), EGFP siRNA (EGFP group, *n* = 10), SCR siRNA alone (SCR group-scramble, noncoding siRNA, *n* = 8), HI-PEMF (*n* = 8), or EP (*n* = 6), as well as in combination with electrotransfer after electroporation by HI-PEMF (*n* = 12) or by conventional EP (EGFP+GET, *n* = 8; SCR-GET, *n* = 7). The data represent the mean and standard error of the mean. Red arrows indicate the day on which gene electrotransfer was performed. * *p* -value < 0.05, significantly different from all other groups except EGFP+GET group; ** *p*-value < 0.05, significantly different from all other groups; ^#^ *p*-value < 0.05, significantly different from all other groups except EGFP+HI-PEMF group; ^+^ *p*-value < 0.05, significantly different from SCR+HI-PEMF group and SRC+GET group. (**B**) For each excised tumor, the fluorescent area was measured on day 6, i.e., two days after the last intratumoral injection of either siMAX universal buffer or siRNA EGFP or siRNA SCR alone or in combination with the gene electrotransfer mediated by HI-PEMF. For the normalization of the fluorescent tumor area and tumor volume, groups were normalized as follows: HI-PEMF or EP treated tumors were normalized to the control; siRNA EGFP treated tumors were normalized to the group siRNA SCR treated tumors; tumors treated with HI-PEMF and siRNA EGFP were normalized to the group HI-PEMF and siRNA SCR treated tumors; tumors treated with EP and siRNA EGFP were normalized to the group EP and siRNA SCR treated tumors. The data represent the mean and standard error of the mean. Each group consisted of 6 to 12 tumors, as described under (A). * *p*-value < 0.05, significantly different from all other groups. (**C**) Representative images of the EGFP fluorescence in melanoma tumors observed on day 6, that is, 2 days after the last intratumoral injection of either siMAX universal buffer or EGFP siRNA or SCR siRNA alone or in combination with HI-PEMF-induced gene electrotransfer. BF—bright field; FL—fluorescence. Scale bar represents 1 mm. (**D**) Representative images of the tumors after immunohistologically staining for proliferation, apoptosis and immune cell (Ki-67, caspase 3, granzyme B). Brown staining represents proliferative cells, caspase 3 positive cells, and granzyme B positive cells. Scale bar represents 50 µm. Each group consisted of 3 tumors.

The silencing effect of EGFP in B16F10 tumors stably expressing EGFP after siRNA EGFP electrotransfer by HI-PEMF in time was also noninvasively assessed by fluorescence stereomicroscopy imaging before and after the treatment on each consecutive day. Three consecutive treatments of tumors with the combination of injection of EGFP siRNA followed by electroporation by HI-PEMF were performed on days 0, 2, and 4 (Figure 4). The extent and time course of the silencing effect of siRNA was determined as the fluorescent tumor area after the therapy normalized to day 0 before therapy. In all control groups, 8 h after the therapy, the fluorescent tumor area stayed at the same level as observed before therapy and increased with time due to the tumor growth. A significant silencing effect of EGFP siRNA using HI-PEMF was obtained 8 h after each gene electrotransfer (up to a 28% smaller fluorescent tumor area); silencing was prolonged up to 2 days only after the third treatment (Figure 4A). Nevertheless, the more pronounced silencing effect of EGFP was observed using conventional EP in a similar time-dependent pattern that reduced the fluorescent tumor area 8 h after each electrotransfer up to 50%. However, the silencing effect using only HI-PEMF or conventional EP was insignificant at day 5 and became significant at day 6, most probably due to the induced cell death after conventional EP. In addition, the treatment of tumors with SCR electrotransferred either with HI-PEMF or conventional EP resulted in a significantly smaller reduction of the fluorescent tumor area compared to electrotransfer of EGFP. Tumors were also excised and imaged without the skin 48 h after the last treatment, i.e., on day 6 (up to a 61% smaller fluorescent tumor area) (Figure 4B,C). Similarly to the results for the noninvasive in vivo imaging, we observed a significant decrease in the fluorescent area of excised tumors when EGFP silencing with HI-PEMF treatment was performed. These results indicate that HI-PEMF enabled the successful electrotransfer of EGFP siRNA molecules into tumor cells.

### 3.3. Histological Analyses

Histological analyses were performed 2 days after the third siRNA EGFP electrotransfer by HI-PEMF. The necrotic area was mainly located in the center of the tumor and there was no difference between the control and treated groups (control: 25.3% ±4.1; HI-PEMF 27.1% ±2.3; EGFP+HI-PEMF: 19.5% ±3.3; EP 16.5% ±2.9; EGFP+GET 13.5% ±1.6). In the viable parts of the tumor, treatments with HI-PEMF, conventional EP or siRNA EGFP electrotransfer by HI-PEMF had no significant effect on proliferation, apoptosis and immune cell infiltration compared to the control (Figure 3D). All tumors were highly proliferative (control 91.7% ±2.8; HI-PEMF 90.4% ±1.9; EGFP+HI-PEMF 89.6% ±1.5, EP 83.3% ±2.1). Furthermore, in tumors after siRNA EGFP electrotransfer by HI-PEMF, a nonsignificant higher increase in apoptosis up to 7% (control 9.3% ±1.2; HI-PEMF 11.1% ±0.8; EGFP+HI-PEMF 16.2% ±0.7; EP 13.4% ±2.7) and up to 4% increase in the number of infiltrating immune cells (control 4.7% ±0.9; HI-PEMF 5.2% ±0.5; EGFP+HI-PEMF 9.1% ±1.5; EP 8.3% ±0.9) were determined in comparison to control. The electrotransfer of siRNA EGFP by conventional electroporation significantly decreased proliferation (EGFP+GET 78.6% ±1.8) and increased the apoptosis (EGFP+GET 19.9% ±2.1) compared to the control but has no significant effect on immune cell infiltration (EGFP+GET 10.1 ±0.4). These data indicate that the electrotransfer of siRNA by HI-PEMF targeting reporter gene EGFP did not induce any morphological changes or considerable immune cell infiltration. However, the electrotransfer EGFP siRNA by conventional EP lead to an increase in apoptotic cell death in tumors.

## 4. Discussion

In our study, we demonstrated contactless high intensity pulsed electromagnetic field (HI-PEMF) treatment as a feasible approach to achieve in vivo transfection in tumors. We show that HI-PEMF enhanced the transport of short siRNA EGFP, resulting in the significant silencing of EGFP in mouse melanoma tumors stably expressing EGFP at mRNA and protein levels and by live fluorescence imaging in vivo. Even though HI-PEMF is currently less efficient than the conventional gene electrotransfer that utilizes either contact or invasive electrodes, we have proved its effectiveness in the transfection of melanoma tumors with siRNA. The proposed technique may thus represent an important tool in research and clinical applications for noninvasive drug and gene/nucleic acid delivery in vivo, since HI-PEMF is easy to perform and is both noninvasive and contactless. 

siRNA delivery is a promising therapeutic approach in cancer therapy, in particular, inactivating the oncogenes and tumor suppressor genes involved in cancer disease [32]. The efficiency of gene silencing depends on the activity of the siRNA delivery to target cells and the activity of siRNA, which can be improved by chemical modifications and optimization of sites and sequences [33]. However, being unstable in the tissues and blood, the effectiveness of siRNA delivery represents the main challenge in the development of this approach. Due to toxicities associated with viral carriers, non-viral carriers, i.e., nanoparticles (of organic or inorganic origin, or a combination of both in hybrid nanoparticles), are most commonly used to deliver siRNA in tumors, which shows a very promising therapeutic approach for cancer treatment at the level of interference with metastases, chemo-resistance of tumors, angiogenesis, and proliferation of tumor cells in preclinical tumor models and in the clinical trials [34,35,36]. Furthermore, the association of siRNA with magnetic nanoparticles was demonstrated for efficient siRNA delivery [37,38]. However, the construction of such carriers must fulfill the precise requirements for the safety, pharmacokinetics, and biodistribution and selectivity of siRNA in the target tissue. Thus, the local delivery approach, i.e., electroporation, may have advantages over systemic delivery in accessible tumors. Successful gene silencing in different cells and tissues was obtained by using conventional electroporation as non-viral delivery approach of siRNA targeting reporter or therapeutic genes [5,21,39,40,41,42,43,44]. Silencing therapeutic CD105 (endoglin), VEGF and Rho GTPase Rac1 genes demonstrated good antitumor and antivascular effects [5,21,45]. The results of these studies indicated that to achieve good transfection efficiency in tumors, multiple intratumoral administrations of therapeutic molecules have to be performed. Although conventional electroporation, i.e., electric pulse-mediated electrotransfer, is inexpensive and simple, it has several limitations and disadvantages, such as the narrow range of clinically safe electric field parameters, the mandatory contact or even invasiveness of electrodes with the tissue, and the possible undesired tissue ablation by irreversible electroporation [46]. 

Already in 2012, Kardos et al. demonstrated the use of HI-PEMF for electrotransfer of plasmid DNA encoding EGFP to guinea pig skin by a magnetic field of 4 T [28]. They showed an approximately 5-fold increase in EGFP expression compared to that for plasmid DNA treatment only. Furthermore, compared to conventional gene electrotransfer, 50 bipolar pulses for a total of 5 s yielded almost equally high levels of EGFP expression. In our study, we applied an approximately 7-times lower time-varying magnetic field than that used by Kardos et al. to evaluate the delivery of approximately 200-times smaller molecules, i.e., 21 base pairs long siRNA molecules. Indeed, significant silencing of EGFP in B16F10 melanoma tumor cells stably expressing EGFP was obtained 8 h after transfection, and the fluorescent area of the tumors decreased by as much as 40% compared to the control. Thereafter, the fluorescent tumor area started to increase, albeit to a lesser extent than that of the pertinent controls, presumably due to increased tumor growth, degradation (short-lived siRNA) of the siRNA, and/or delivery of the siRNA to only a fraction of the tumor cells inside the tumor. The silencing effect of EGFP was however more pronounced when using conventional electroporation (by 77% compared to the control) compared to HI-PEMF, as the local electric field in the target tissue during conventional electroporation was considerably higher (4000 times) compared to the induced field by HI-PEMF. Similar findings were obtained by Golzio et al. using conventional electroporation for electrotransfer of siRNA against EGFP [43]. They obtained a significant decrease of up to 30% in the EGFP fluorescence of the B16F10 melanoma stably expressing an EGFP tumor within 2 to 4 days following a single gene electrotransfer of GFP22 siRNA. Additionally, a noticeable increase in the EGFP fluorescence level was observed at day 5, due to short-lived siRNAs. The decrease of EGFP fluorescence in tumors correlated with the reduction of EGFP expression at the mRNA level by 50% and by 60% at the protein level. In order to improve the silencing effect, we repeated conventional electrotransfer of siRNA against EGFP in melanoma tumors three times and compared it with the silencing effect of HI-PEMF electrotransfer that was also applied three times. However, similar results to the study of Golzio et al. were obtained in our conventional electrotransfer protocol, EGFP mRNA was degraded for 61% and EGFP protein expression was reduced for 63%. This suggested that the partial silence of EGFP in the tumor was due to the short lifetime of transferred siRNA in the cytoplasm and low transfection efficiency. The electrotransfer of siRNA with HI-PEMF was significantly less efficient; up to 22% on the mRNA and protein level compared to conventional gene electrotransfer. However, the induced electric field established by HI-PEMF was more than 4000 times lower compared to the local electric field in conventional GET. Despite lower efficiency, we confirmed the proof of concept for noninvasive electrotransfer of EGFP siRNA molecules into tumor cells by means of HI-PEMF. 

Although nonviral delivery of genetic material into cells using conventional electroporation holds great promise for basic research and clinics, it has some limitations, i.e., low transfection efficiency compared to that of other methods [47,48,49] and difficulties in controlling the delivery, due to an incomplete understanding of its mechanisms [50]. In gene electrotransfer using conventional electroporation of cells and tissues, it is postulated that once the cell membrane is permeabilized and the membrane-plasmid DNA complex is formed, the DNA passes through the membrane, where it is transported to the nucleus [50,51,52,53]. The formation of membrane-plasmid DNA complex and transport across the membrane is facilitated by electrophoretic force excited by an electric pulse. Such processes demand optimized electric pulse parameters, namely, longer, high voltage or a combination of short, high voltage and long, low voltage pulses [52,54,55,56]. It has also been shown that the size and charge of molecules influence their passage through the cell membrane after electroporation [57]. While small molecules can enter cells presumably through “the electropores” formed in the membrane, the transport of macromolecules such as plasmid DNA is more complex [58,59]. Specifically, plasmid DNA can enter through pores only partially, and it was recently shown that mainly clathrin-mediated or Rac-1-dependent endocytosis processes are responsible for the transport of plasmid DNA across the membrane and cytoplasm into the cell [53]. Furthermore, the alterations in the pH of the medium during electroporation may cause the denaturation of the plasmid DNA and thus play an important role in efficient DNA electrotransfer [60,61]. Additionally, the temperature of the cell membrane affects its fluidity and consequently, its electroporation. It was shown that gene electrotransfer could be thermally assisted. Specifically, heating applied prior to electroporation enabled a similar level of transfection efficiency in the skin using an approximately 30% lower electric field [62]. In our study, we observed an increase in the temperature of the magnetic coil (approximately 40 °C, data not shown), which could contribute to the transfection efficiency. In addition, the heterogeneity of tissue, i.e., the cell type organization and extracellular matrix composition, has a substantial impact on transfection efficiency. It was shown that the modification of the tissue extracellular matrix improved the transfection efficiency, due to improved plasmid DNA distribution within the tissue [20]. In summary, we are aware of all these factors that might have affected the transfection efficiency in our study and intend to address these potential contributions in future studies by optimizing the conditions during HI-PEMF and improving the equipment, i.e., the coil and magnetic pulse generator, to reduce heating and/or heat transfer.

In cancer treatments, immunostimulation is an important component of the treatment. An siRNA can elicit an immune response and immunostimulatory activities of siRNA may represent novel agents for treating cancers [63]. Recently, it has been shown that the induction of immunogenic cell death by electrochemotherapy and/or boosting the immune response by IL-12 or TNFα plasmid DNA electrotransfer, both using conventional electroporation as a delivery approach, resulted in a good antitumor effect in murine tumor models [9,10,31,64]. In the present study, the EGFP siRNA electrotransfer by conventional electroporation or by HI-PEMF did not elicit significant immune cell infiltration. However, the conventional electroporation significantly increased the apoptotic death of tumor cells that correlated with considerable tumor growth delay, while this was not evident after silencing mediated by HI-PEMF. The introduction of foreign DNA or RNA is known to elicit the pattern-recognition receptors that lead to the activation of immune response and cell death [65]. Recently, an increased apoptotic cell death of tumor cells after the conventional electrotransfer of different control plasmids DNA without therapeutic gene due to activation of cytosolic DNA sensors that can trigger an immune response was shown [66]. Thus, the silencing effect observed after electrotransfer of SCR siRNA by HI-PEMF or conventional electroporation suggests to be induced by increased tumor cell death. 

The primary goal of our study was to demonstrate HI-PEMF treatment as a feasible approach to achieve in vivo transfection in tumors. Thus, we can speculate, that targeting therapeutic genes with specific engineered immunomodulatory siRNA delivered by HI-PEMF, could be used for modulation of the immune response. Recent studies indicated a promising therapeutic efficiency in cancer with a silencing approach for intracellular negative immune regulators in tumors, i.e., IDO1, PD-1 or PD-L1 [67,68,69]. Several approaches were used for delivery of siRNA against IDO1 or PD-L1 in immune cells (dendritic cells, T cells): intramuscular, intradermal, intravenous injection; biolistic device; oral application of attenuated bacteria; synthetic polymers, nanoparticles and viral vectors [67,68]. As a result of silencing IDO1, PDL-1 an activation of immune response was determined in several cancer models (breast, bladder, colon, orthotopic and metastatic liver cancer, melanoma) showing good antitumor response. For this purposes HI-PEMF has the potential for clinical application since it is both noninvasive and contactless.

We have already shown that small molecules can be delivered with HI-PEMF in vitro [26] and in vivo [29]. Few studies have evaluated the mechanisms of molecule uptake after the exposure of cells to HI-PEMF. As for conventional electroporation, it was shown that the pores formed in the membrane quickly resealed [70] and endocytosis was needed as the transport mechanism of molecules for electroporation at lower electric field strengths [25]. We suspect that the exposure of tissues to the magnetic field of 1 T did not induce an electric field typically required for electroporation, as its amplitude was approximately 1000 times lower. Nevertheless, we believe HI-PEMF delivery approach can be improved and optimized for transferring also bigger molecules, such as plasmid DNA encoding therapeutic molecules in different tissues appropriate for immunotherapy, i.e., skin and muscle. Although the exact mechanism of HI-PEMF inducing transport of siRNA to tumor cells is currently unknown, we work under the assumption that the induced electric field and membrane electroporation are the principal mechanisms [58]. Since HI-PEMF used in electrochemotherapy has already proved successful, it could be used as conventional electrochemotherapy for immune-boosting and also in the gene transfer of therapeutic targets for immunomodulation and in vaccination protocols.

## 5. Conclusions

Our results show that HI-PEMF at a magnetic field below 1 T is sufficient to achieve the transfection of tumor cells because small molecules of siRNA against EGFP were delivered to tumors. Due to the simple and contactless application of HI-PEMF, this approach represents a potential alternative to conventional electroporation. Further studies are thus warranted to improve the equipment, optimize the protocols for genetic material application and the HI-PEMF parameters, and demonstrate the effects of HI-PEMF on a broader range of different tissues. 

## References

[1] Chang D.C., Reese T.S. (1990). Changes in membrane structure induced by electroporation as revealed by rapid-freezing electron microscopy. Biophys. J..

[2] Kotnik T., Rems L., Tarek M., Miklavčič D. (2019). Membrane Electroporation and Electropermeabilization: Mechanisms and Models. Annu. Rev. Biophys..

[3] Neumann E., Schaefer-Ridder M., Wang Y., Hofschneider P.H. (1982). Gene transfer into mouse lyoma cells by electroporation in high electric fields. EMBO J..

[4] Kos S., Blagus T., Cemazar M., Lampreht Tratar U., Stimac M., Prosen L., Dolinsek T., Kamensek U., Kranjc S., Steinstraesser L. (2016). Electrotransfer parameters as a tool for controlled and targeted gene expression in skin. Mol. Ther. Nucleic Acids.

[5] Dolinsek T., Markelc B., Sersa G., Coer A., Stimac M., Lavrencak J., Brozic A., Kranjc S., Cemazar M. (2013). Multiple delivery of siRNA against endoglin into murine mammary adenocarcinoma prevents angiogenesis and delays tumor growth. PLoS ONE.

[6] Heller L.C., Heller R. (2010). Electroporation Gene Therapy Preclinical and Clinical Trials for Melanoma. Curr. Gene Ther..

[7] Vasan S., Hurley A., Schlesinger S.J., Hannaman D., Gardiner D.F., Dugin D.P., Boente-Carrera M., Vittorino R., Caskey M., Andersen J. (2011). In Vivo Electroporation Enhances the Immunogenicity of an HIV-1 DNA Vaccine Candidate in Healthy Volunteers. PLoS ONE.

[8] Yarmush M.L., Golberg A., Serša G., Kotnik T., Miklavčič D. (2014). Electroporation-based technologies for medicine: Principles, applications, and challenges. Annu. Rev. Biomed. Eng..

[9] Sersa G., Teissie J., Cemazar M., Signori E., Kamensek U., Marshall G., Miklavcic D. (2015). Electrochemotherapy of tumors as in situ vaccination boosted by immunogene electrotransfer. Cancer Immunol. Immunother..

[10] Shi G., Edelblute C., Arpag S., Lundberg C., Heller R. (2018). IL-12 Gene Electrotransfer Triggers a Change in Immune Response within Mouse Tumors. Cancers.

[11] Calvet C.Y., Mir L.M. (2016). The promising alliance of anti-cancer electrochemotherapy with immunotherapy. Cancer Metastasis Rev..

[12] Mali B., Jarm T., Snoj M., Sersa G., Miklavcic D. (2013). Antitumor effectiveness of electrochemotherapy: A systematic review and meta-analysis. Eur. J. Surg. Oncol..

[13] Campana L.G., Marconato R., Valpione S., Galuppo S., Alaibac M., Rossi C.R., Mocellin S. (2017). Basal cell carcinoma: 10-year experience with electrochemotherapy. J. Transl. Med..

[14] Tozon N., Lampreht Tratar U., Znidar K., Sersa G., Teissie J., Cemazar M. (2016). Operating procedures of the electrochemotherapy for treatment of tumor in dogs and cats. J. Vis. Exp..

[15] Kotnik T., Kramar P., Pucihar G., Miklavcic D., Tarek M. (2012). Cell membrane electroporation—Part 1: The phenomenon. IEEE Electr. Insul. Mag..

[16] Weaver J.C. (1995). Electroporation in cells and tissues: A biophysical phenomenon due to electromagnetic fields. Radio Sci..

[17] Wagstaff P.G.K., Buijs M., van den Bos W., de Bruin D.M., Zondervan P.J., de la Rosette J.J.M.C.H., Laguna Pes M.P. (2016). Irreversible electroporation: State of the art. Onco. Targets. Ther..

[18] Golzio M., Mora M.P., Raynaud C., Delteil C., Teissié J., Rols M.P. (1998). Control by osmotic pressure of voltage-induced permeabilization and gene transfer in mammalian cells. Biophys. J..

[19] Maglietti F., Michinski S., Olaiz N., Castro M., Suárez C., Marshall G. (2013). The Role of Ph Fronts in Tissue Electroporation Based Treatments. PLoS ONE.

[20] Cemazar M., Golzio M., Sersa G., Escoffre J.-M.M., Coer A., Vidic S., Teissie J. (2012). Hyaluronidase and Collagenase Increase the Transfection Efficiency of Gene Electrotransfer in Various Murine Tumors. Hum. Gene Ther..

[21] Takei Y., Nemoto T., Mu P., Fujishima T., Ishimoto T., Hayakawa Y., Yuzawa Y., Matsuo S., Muramatsu T., Kadomatsu K. (2008). In vivo silencing of a molecular target by short interfering RNA electroporation: Tumor vascularization correlates to delivery efficiency. Mol. Cancer Ther..

[22] Dini L., Dwikat M., Panzarini E., Vergallo C., Tenuzzo B. (2009). Morphofunctional study of 12-O-tetradecanoyl-13-phorbol acetate (TPA)-induced differentiation of U937 cells under exposure to a 6 mT static magnetic field. Bioelectromagnetics.

[23] Bodega G., Forcada I., Suárez I., Fernández B. (2005). Acute and chronic effects of exposure to a 1-mT magnetic field on the cytoskeleton, stress proteins, and proliferation of astroglial cells in culture. Environ. Res..

[24] Flipo D., Fournier M., Benquet C., Roux P., Le Boulaire C., Pinsky C., LaBella F.S., Krzystyniak K. (1998). Increased apoptosis, changes in intracellular Ca2+, and functional alterations in lymphocytes and macrophages after in vitro exposure to static magnetic field. J Toxicol Env. Heal. A.

[25] Towhidi L., Firoozabadi S.M.P., Mozdarani H., Miklavcic D. (2012). Lucifer Yellow uptake by CHO cells exposed to magnetic and electric pulses. Radiol. Oncol..

[26] Novickij V., Dermol J., Grainys A., Kranjc M., Miklavčič D. (2017). Membrane permeabilization of mammalian cells using bursts of high magnetic field pulses. PeerJ.

[27] Novickij V., Grainys A., Svedienė J., Markovskaja S., Paškevičius A., Novickij J. (2014). Microsecond pulsed magnetic field improves efficacy of antifungal agents on pathogenic microorganisms. Bioelectromagnetics.

[28] Kardos T.J., Rabussay D.P. (2012). Contactless magneto-permeabilization for intracellular plasmid DNA delivery in-vivo. Hum. Vaccin. Immunother..

[29] Kranjc S., Kranjc M., Scancar J., Jelenc J., Sersa G., Miklavcic D. (2016). Electrochemotherapy by pulsed electromagnetic field treatment (PEMF) in mouse melanoma B16F10 in vivo. Radiol. Oncol..

[30] Caplen N.J., Parrish S., Imani F., Fire A., Morgan R.A. (2001). Specific inhibition of gene expression by small double-stranded RNAs in invertebrate and vertebrate systems. Proc. Natl. Acad. Sci..

[31] Savarin M., Kamensek U., Cemazar M., Heller R., Sersa G. (2017). Electrotransfer of plasmid DNA radiosensitizes B16F10 tumors through activation of immune response. Radiol. Oncol..

[32] Tatiparti K., Sau S., Kashaw S., Iyer A. (2017). siRNA Delivery Strategies: A Comprehensive Review of Recent Developments. Nanomaterials.

[33] Hassler M.R., Turanov A.A., Alterman J.F., Haraszti R.A., Coles A.H., Osborn M.F., Echeverria D., Nikan M., Salomon W.E., Roux L. (2018). Comparison of partially and fully chemically-modified siRNA in conjugate-mediated delivery in vivo. Nucleic Acids Res..

[34] Kasai H., Inoue K., Imamura K., Yuvienco C., Montclare J.K., Yamano S. (2019). Efficient siRNA delivery and gene silencing using a lipopolypeptide hybrid vector mediated by a caveolae-mediated and temperature-dependent endocytic pathway. J. Nanobiotechnology.

[35] Akhtar S., Benter I.F. (2007). Nonviral delivery of synthetic siRNAs in vivo. J. Clin. Invest..

[36] Watts J.K., Corey D.R. (2012). Silencing disease genes in the laboratory and the clinic. J. Pathol..

[37] Ensenauer R., Hartl D., Vockley J., Roscher A., Fuchs U. (2011). Efficient and gentle siRNA delivery by magnetofection. Biotech. Histochem..

[38] Singh J., Mohanty I., Rattan S. (2018). In vivo magnetofection: A novel approach for targeted topical delivery of nucleic acids for rectoanal motility disorders. Am. J. Physiol. Liver Physiol..

[39] Keller A.-A., Maeß M.B., Schnoor M., Scheiding B., Lorkowski S. (2018). Transfecting Macrophages. Methods Mol. Biol..

[40] Lojk J., Mis K., Pirkmajer S., Pavlin M. (2015). siRNA delivery into cultured primary human myoblasts-optimization of electroporation parameters and theoretical analysis. Bioelectromagnetics.

[41] Huang D., Zhao D., Wang X., Li C., Yang T., Du L., Wei Z., Cheng Q., Cao H., Liang Z. (2018). Efficient delivery of nucleic acid molecules into skin by combined use of microneedle roller and flexible interdigitated electroporation array. Theranostics.

[42] Takahashi Y., Nishikawa M., Kobayashi N., Takakura Y. (2005). Gene silencing in primary and metastatic tumors by small interfering RNA delivery in mice: Quantitative analysis using melanoma cells expressing firefly and sea pansy luciferases. J. Control. Release.

[43] Golzio M., Mazzolini L., Ledoux A., Paganin A., Izard M., Hellaudais L., Bieth A., Pillaire M.J., Cazaux C., Hoffmann J.S. (2007). In vivo gene silencing in solid tumors by targeted electrically mediated siRNA delivery. Gene Ther..

[44] Wei Z., Huang Y., Zhao D., Hu Z., Li Z., Liang Z. (2015). A pliable electroporation patch (ep-Patch) for efficient delivery of nucleic acid molecules into animal tissues with irregular surface shapes. Sci. Rep..

[45] Vader P., van der Meel R., Symons M.H., Fens M.H.A.M., Pieters E., Wilschut K.J., Storm G., Jarzabek M., Gallagher W.M., Schiffelers R.M. (2011). Examining the role of Rac1 in tumor angiogenesis and growth: A clinically relevant RNAi-mediated approach. Angiogenesis.

[46] Rubinsky L., Guenther E., Mikus P., Stehling M., Rubinsky B. (2016). Electrolytic Effects During Tissue Ablation by Electroporation. Technol. Cancer Res. Treat..

[47] Kim S.I., Shin D., Lee H., Ahn B.Y., Yoon Y., Kim M. (2009). Targeted delivery of siRNA against hepatitis C virus by apolipoprotein A-I-bound cationic liposomes. J. Hepatol.

[48] Vaseghi G., Rafiee L., Javanmard S.H. (2017). Non-viral Delivery Systems for breast cancer gene therapy. Curr. Gene Ther..

[49] Wilson R.C., Gilbert L.A. (2018). The Promise and Challenge of In Vivo Delivery for Genome Therapeutics. ACS Chem. Biol..

[50] Wu M., Yuan F. (2011). Membrane binding of plasmid DNA and endocytic pathways are involved in electrotransfection of mammalian cells. PLoS ONE.

[51] Rosazza C., Meglic S.H., Zumbusch A., Rols M.-P., Miklavcic D. (2016). Gene Electrotransfer: A Mechanistic Perspective. Curr. Gene Ther..

[52] Haberl S., Miklavčič D., Serša G., Frey W., Rubinsky B. (2013). Cell membrane electroporation—Part 2: The applications. IEEE Electr. Insul. Mag..

[53] Mao M., Wang L., Chang C.C., Rothenberg K.E., Huang J., Wang Y., Hoffman B.D., Liton P.B., Yuan F. (2017). Involvement of a Rac1-Dependent Macropinocytosis Pathway in Plasmid DNA Delivery by Electrotransfection. Mol. Ther..

[54] Satkauskas S. (2002). Mechanisms of in Vivo DNA Electrotransfer: Respective Contributions of Cell Electropermeabilization and DNA Electrophoresis. Mol. Ther..

[55] André F.M., Gehl J., Sersa G., Préat V., Hojman P., Eriksen J., Golzio M., Cemazar M., Pavselj N., Rols M.-P. (2008). Efficiency of High- and Low-Voltage Pulse Combinations for Gene Electrotransfer in Muscle, Liver, Tumor, and Skin. Hum. Gene Ther..

[56] Cemazar M., Golzio M., Sersa G., Hojman P., Kranjc S., Mesojednik S., Rols M.P., Teissie J. (2009). Control by pulse parameters of DNA electrotransfer into solid tumors in mice. Gene Ther..

[57] Sözer E.B., Wu Y.H., Romeo S., Vernier P.T. (2017). Nanometer-Scale Permeabilization and Osmotic Swelling Induced by 5-ns Pulsed Electric Fields. J. Membr. Biol..

[58] Breton M., Delemotte L., Silve A., Mir L.M., Tarek M. (2012). Transport of siRNA through lipid membranes driven by nanosecond electric pulses: An experimental and computational study. J. Am. Chem. Soc..

[59] Sachdev S., Feijoo Moreira S., Keehnen Y., Rems L., Kreutzer M.T., Boukany P.E. (2019). DNA-membrane complex formation during electroporation is DNA size-dependent. Biochim. Biophys. Acta Biomembr..

[60] Turjanski P., Olaiz N., Maglietti F., Michinski S., Suárez C., Molina F.V., Marshall G. (2011). The role of pH fronts in reversible electroporation. PLoS ONE.

[61] Potočnik T., Miklavčič D., Maček Lebar A. (2019). Effect of electroporation and recovery medium pH on cell membrane permeabilization, cell survival and gene transfer efficiency in vitro. Bioelectrochemistry.

[62] Donate A., Bulysheva A., Edelblute C., Jung D., Malik M.A., Guo S., Burcus N., Schoenbach K., Heller R. (2016). Thermal Assisted In Vivo Gene Electrotransfer. Curr. Gene Ther..

[63] Meng Z., Lu M. (2017). RNA Interference-Induced Innate Immunity, Off-Target Effect, or Immune Adjuvant?. Front. Immunol..

[64] Kamensek U., Cemazar M., Lampreht Tratar U., Ursic K., Sersa G. (2018). Antitumor in situ vaccination effect of TNFα and IL-12 plasmid DNA electrotransfer in a murine melanoma model. Cancer Immunol. Immunother..

[65] Desmet C.J., Ishii K.J. (2012). Nucleic acid sensing at the interface between innate and adaptive immunity in vaccination. Nat. Rev. Immunol..

[66] Bosnjak M., Jesenko T., Kamensek U., Sersa G., Lavrencak J., Heller L., Cemazar M. (2018). Electrotransfer of Different Control Plasmids Elicits Different Antitumor Effectiveness in B16.F10 Melanoma. Cancers.

[67] Zhang M., Liu K., Wang M. (2019). Development of cancer immunotherapy based on PD-1/PD-L1 pathway blockade. RSC Adv..

[68] Liu Y.-H., Yeh I.-J., Lai M.-D., Liu K.-T., Kuo P.-L., Yen M.-C. (2019). Cancer Immunotherapy: Silencing Intracellular Negative Immune Regulators of Dendritic Cells. Cancers.

[69] Wu Y., Gu W., Li J., Chen C., Xu Z.P. (2019). Silencing PD-1 and PD-L1 with nanoparticle-delivered small interfering RNA increases cytotoxicity of tumor-infiltrating lymphocytes. Nanomedicine.

[70] Liu F., Heston S., Shollenberger L.M., Sun B., Mickle M., Lovell M., Huang L. (2006). Mechanism of in vivo DNA transport into cells by electroporation: Electrophoresis across the plasma membrane may not be involved. J. Gene Med..

